# Assessment of Health Level and Socio-Economic Characteristics of People Working in the Shipbuilding Industry: A Control Group Study

**DOI:** 10.5539/gjhs.v7n2p154

**Published:** 2014-10-08

**Authors:** Agoritsa Koulouri, Zoe Roupa, Pavlos Sarafis, Chryssi Hatzoglou, Konstantinos Gourgoulianis

**Affiliations:** 1 Health Center of Salamina, Greece; 2 Department of Life & Health Sciences, University of Nicosia, Cyprus; 3 Department of Nursing, Technological Institution of Sterea Ellada, Greece; 4 Department of Medicine, University of Thessaly, Larisa, Greece

**Keywords:** health level, self-reported health, unemployment, social- economic status

## Abstract

**Introduction:**

The health level of the population and the way people perceive it has been associated with their physical and mental health, as well as with their social and occupational characteristics.

**Purpose:**

The comparative assessment of mental and health level in shipbuilding industry workers and general population and its relationship to social and economic parameters.

**Methods:**

A group of one hundred men working in the shipbuilding industry aged 51.8±8.2 years old and a control group of one hundred men of the general population aged 51.1±6.4 were studied. All participants completed the General Health Questionnaire – 28 and Fagerstrom test and a form with demographic, occupational and economic status characteristics. The statistical software SPSS 17.0 was used for data analysis.

**Results:**

Twenty–six percent of the general population and 47% of men working in the shipbuilding industry assessed their health as moderate/poor. Higher median values of anxiety and depressive symptomatology were observed in individuals characterizing their health as moderate/poor (p<0.001), their work as physically too demanding and in individuals with high dependency on smoking (p<0.05). With regard to the parameter of physical complaints, people working in the shipbuilding industry, non-active employees and those with comorbidities were found more burdened in relation to the general population (p<0.05). Depressive disorders were more common in those stating that their economic situation had been significantly deteriorated and in individuals with chronic diseases, which also showed reduced social functioning (p<0.05).

**Conclusions:**

Health level and its individual dimensions are both associated with health self-assessment and occupational and economic status. The coexistence of chronic diseases and smoking dependence affects emotion and social functioning of individuals.

## 1. Introduction

The most important determinants of health and quality of life concern not only the socio-economic conditions but also the physical and psychosocial health of the individual (Yfantopoulos & Sarris, 2001). The level of health as perceived by the individuals themselves, although based on the actual status of their health, does not necessarily coincide with it (Sadana et al., 2000). Also, socio-economic and psychological circumstances and harmful habits in one’s life are reflected in how the person evaluates his/her health level (Idler & Kasl, 1995).

Social functioning is defined as the individual’s ability to perform the obligations of everyday life, to show beneficial social activity, to be able to cooperate and develop skills that enable him/her to create and maintain relationships and communication with others and it plays an important role in assessing health level. Expectations and demands regarding health level differ from person to person, depending on individual’s identity and culture, his/her cultural aspects, physical problems, the possession of specific knowledge and characteristics that shape individual’s perceptions (Sadana et al., 2000).

The identification of individuals with psychiatric symptomatology and mood disorders in the community contributes to the assessment of the factors determining the use of Health Services, the incidence of specific psychiatric disorders and to investigate the correlation of psychiatric symptoms to social, demographic and economic factors (Thornicroft, 2001).

Anxiety and depressive disorders and their symptoms significantly affect the actions of the individual in a large number of fields such as occupational, social, and health (Cleland, Lee, & Hall, 2007). The quality of life in depressed patients is affected by the disease and is more burdensome when coexisting with diabetes, hypertension or other chronic respiratory diseases (Martin et al., 2008).

Scientific research internationally and in our country has shown that the economic crisis causes serious health problems in the population (Kyriopoulos & Tsiantou, 2010). According to the study of the Organisation for Economic Co-operation and Development (OECD) in 2012, the economic crisis has led to an increase in poverty, unemployment and anxiety, which are associated with worse health outcomes.

Duration of work activity and age are strong determinants of mental problems incidence. Mental pressure and anxiety are significantly affected by low job satisfaction, workload and negative characteristics of the workplace.

The purpose of this study was the comparative assessment of mental and physical health level in people working in the shipbuilding industry and the general population in the community. Mental and physical health relation to social, economic, employment and demographic parameters in both groups was also investigated.

## 2. Method

A group of one hundred people working in the Shipbuilding Industry (SI) was studied along with a control group of one hundred people of the general population. All participants completed General Health Questionnaire – 28 and Fagerstrom test and a form in which they recorded demographic, occupational and economic status characteristics. The questionnaires were completed after prior information about the purposes of the study and obtain the consent of the participants. All were middle aged men, integrated into the labor force and constituted a convenience sample.

### 2.1 Measurement Tools

Data were collected by using a questionnaire with two sections. The first section was a form with demographic, medical history and occupational status data. The second section included the General Health Assessment questionnaire (General health Questionnaire 28), an international standard for the labeling of milder forms of psychopathology in the general population. The questionnaire was translated into Greek with satisfactory reliability and validity indicators of the Greek version (Goldberg et al., 1978; Garyfallos et al., 1991). Item by item and subject by subject results indicate that Greek and English versions are equivalent. Validity coefficients are satisfactory: sensitivity 87%, specificity 85%, misclassification rate 14%. The questions are divided into four subscales concerning physical complaints, anxiety /insomnia, social functioning and depression. The degree of nicotine dependence was assessed by the Fagerstrom test (Heatherton, Kozlowski, Frecker, & Fagerstrom, 1991). Highest score refers to a greater burden.

### 2.2 Ethics

This study was approved by University of Thessaly Ethical Committee (No. 4922/05-11-10).

### 2.3 Statistical Analysis

Student’s t-test and Mann-Whitney nonparametric test were used to compare quantitative variables between two groups. Parametric analysis of variance (ANOVA) or Kruskal-Wallis nonparametric test was used to compare quantitative variables between more than two groups. Bonferroni correction was used to counteract type I error due to multiple comparisons and Pearson’s chi-squared test or Fisher’s exact test was used to compare proportions. The level of statistical significance was set at 0.05 and SPSS 17.0 statistical software was used in the analysis.

## 3. Results

The participants were 100 people of the general population and 100 people working in the Shipbuilding Industry (SI). The average age of both groups was 51.8±8.2 and 51.1±6.4 years with 25.1±11.5 and 25.46±10 years smoking duration, respectively. 73% and 88% were married and 55% and 53% respectively had secondary/higher education.

There was a significantly higher percentage of active employees in the general population (75%) compared with the corresponding percentage in the group of people in the shipbuilding industry (62%). The years of work were 10±3 and 25.4±8.7 (p < 0.001), while the months of unemployment were 24±7 and 13.8±9.7 in the two groups, respectively. 26% of the general population and 47% of those working in the shipbuilding industry assessed their health as moderate/poor, while 10% and 21% respectively had been admitted to hospital in the last year.

In order of frequency, the following diseases were found in the general population: arterial hypertension 12%, Chronic Obstructive Pulmonary Disease (COPD) 10%, heart diseases 9%, orthopedic problems 2%, diabetes mellitus 6% and other diseases 7%. Respectively, the following diseases were found in those working in the shipbuilding industry: arterial hypertension 7%, COPD 13%, heart diseases 4%, orthopedic problems 7%, diabetes mellitus 4% and other diseases 7%.

Smoking dependence was slightly higher in the group of those working in the shipbuilding industry, although there was no statistically significant difference in Fagerstrom score between the two groups (Table 1). High scores in anxiety and depressive symptomatology parameters were observed in individuals who characterized their health as moderate/poor (p<0.001), their work as physically too demanding and in individuals with high smoking dependency. After *post-hoc* analysis (Bonferroni correction), it was found that participants whose work is too demanding had significantly higher scores indicating greater depression compared with participants whose work was quite demanding (p=0.008), as well as those who felt that their economic situation had been significantly deteriorated (p=0.010). Higher scores were also shown by individuals with chronic diseases and high smoking dependence (p=0.003) (Table 2).

**Table 1:** Characteristics of the study population

		**Group**				P Pearson’s x² test

		General population		People working in Shipbuilding Industry		
		N	%	N	%	
**Age. mean score ±SD**		51.8±8.2		51.1±6.4		0.453**
**Years of smoking**		25.1±11.5		25.46±10		0.822
**Married**	No	*27*	*27.0*	*12*	*12.0*	
	Yesι	*73*	*73.0*	*88*	*88.0*	
**Current employment status**	Working	*75*	*75.0*	*62*	*62.0*	0.048**
	Unemployed	*25*	*25.0*	*38*	*38.0*	
**Years of work. mean score ±SD**		10±3		25.4±8.7		**<0.001**-*
**Months of unemployment. mean score ±SD**		24±7		13.8±9.7		**<0.001**-*
**According to you. your health is:**	Moderate/poor	*26*	*26.0*	*47*	*47.0*	***0.002***
	Good / excellent	*74*	*74.0*	*53*	*53.0*	
**Admission to hospital last year**	No	90	90.0	77	78.6	**0.027**
Yes	10	10.0	21	21.4	

		**mean score ±SD**	**Median (int. range)**	**mean score ±SD**	**Median (int. range)**	**P Mann-Whitney**

**FAGERSTROM SCORE**		5.5±2.9	6 (4–8)	6.1±2.5	6 (4–8)	0.306
**Smoking addiction N(%)**						
Low		24 (24.0)		18 (18.0)		0.565**
Moderate		31 (31.0)		35 (35.0)		
High		45 (45.0)		47 (47.0)		

* t test.

** Pearson’s x² test.

**Table 2:** Anxiety, depression and socio-economic characteristics

		Anxiety		Depression	

		Median (IQR)	P Student’s t-test	Median (IQR)	P Mann-Whitney
**Group**	General population	9 (7–12)	0.173	1 (0–4)	0.420
	People working in Shipbuilding Industry	10 (7–14)		2 (0–7)	
**Married**	No	10 (1–13)	0.406	1 (0–5)	0.322
	Yes	10 (7–13)		2 (0–7)	
**Current employment status**	Working	10 (7–13)	0.906	1 (0–6)	0.102
	Unemployed	10 (7–12)		3 (0–7)	
**According to you. your health is:**	Moderate/poor	11 (8–14)	**0.001**	3 (0–8)	**<0.001**
	Good / excellent	9 (5–12)		1 (0–4)	
**Your job is physically demanding:**	None / a little	12 (8–15)	0.009*	1 (0–4)	0.011*
	Enough	9 (6–12)		1 (0–5)	
	Very	10 (7–12)		3 (0–7)	
**Thinking back one year. would you say that the financial situation of your household has :**	Remains the same / improved significantly	9 (7–13)	0.592*	1 (0–3)	0.001*
	It somehow got worse	9.5 (6–12)		1 (0–4)	
	Has significantly worsened	11 (7–13)		5 (0–7.5)	
**Other diseases or chronic complications**	No	9 (5–12)	0.107	1 (0–4)	**0.036**
	Yes	10 (8–13)		3 (0–8)	
**Smoking addiction**	Low	9 (3–12)	0.047*	1 (0–2)	0.008*
	Moderate	10 (8–13)		1.5 (0–4)	
	High	11 (7–13.5)		3 (0–8)	

IQR: Inter Quartile Ratio.

* Kruskal-Wallis test.

Regarding physical complaints those working in the shipbuilding industry (p<0.05) were found severely burdened compared to the general population, individuals who assessed their health as moderate/poor (p<0.001), those who had lost their jobs and individuals with comorbidities (p<0.05). Also, impaired social functioning was observed in inactive employees, in individuals who assessed their health as moderate/poor (p<0.001) and in individuals who suffered from a chronic disease (p<0.05) (Table 3).

**Table 3:** Somatic symptoms, social functioning and socio–economic characteristics

		Physical complaints		Social functioning	

		Median (IQR	P Student’s t-test	Median (IQR	P Mann-Whitney
**Group**	General population	7 (5–10)	**0.002**	8 (7–10)	0.467
	People working in Shipbuilding Industry	9 (6.5–11)		7 (7–9)	
**Married**	No	8 (5–11)	0.648	8 (6–10)	0.901
	Yes	8 (5–11)		8 (7–10)	
**Current employment status**	Working	8 (5–10)	**0.025**	7 (6–9)	**0.002**
	Unemployed	9 (7–11)		8 (7–11)	
**According to you. your health is:**	Moderate/poor	10 (9–11)	**<0.001**	9 (8–11)	**<0.001**
	Good / excellent	7 (4–10)		7 (6–8)	
**Your job is physically demanding:**	None / a little	10 (8–11)	0.005*	8 (6–10)	0.541**
	Enough	8 (5–10)		8 (7–10)	
	Very	7 (4–10)		7 (7–9)	
**Thinking back one year. would you say that the financial situation of your household has :**	Remains the same / improved significantly	9 (7–11)	0.038*	8 (7–10)	0.796**
	It somehow got worse	7 (3–10)		7 (6–10)	
	Has significantly worsened	8 (5–11)		8 (7–10)	
**Other diseases or chronic complications**	No	7 (5–9)	**<0.001**	7 (6–8)	**<0.001**
	Yes	10 (7–11)		8 (7–10)	
**Smoking addiction**	Low	8 (3–10)	0.092*	7 (6–10)	0.474**
	Moderate	8 (5–10)		8 (7–9)	
	High	9 (5.5–11)		8 (7–10)	

IQR: Inter Quartile Ratio.

* ANOVA

** Kruskal-Wallis test.

## 4. Discussion

The present study aimed at a comparative presentation of demographic and socioeconomic characteristics, as well as of the health level dimensions of people working in the shipbuilding industry and in the general population.

Our study showed that higher anxiety and depression were observed in individuals who assessed their health as moderate/poor, in individuals whose work was physically too demanding, had a high smoking dependence and their economic situation had been significantly deteriorated. This is also the conclusion of previous researchers who argue that there is a significant correlation between the economic situation and health level of individuals and that individuals’ income situation is a strong determinant of their health throughout their lifetime (Van Rossum, van de Mheen, Mackenbach, & Grobbee, 2000, Dorsten & Li, 2010; Matthew, Smith, Gariépy, & PhDe, 2013). For this reason, the periods of economic crises, where people’s occupational activity deteriorates and income decreases, are a source of mental and emotional disorders. (Mackenbach, 2005; Malliarou, & Sarafis, 2012).

In this work, the self-assessment of health as moderate or poor was associated with the emergence of anxiety and depression, impaired social functioning and the emergence of physical complaints. The percentage of people in the general population of this research believing that their health is good/excellent reaches 74% and is totally consistent with the findings of a recent research in the European Union (OECD, Society at a Glance., 2012), where it appears that the percentage of Greeks who assess their health as very good or good approaches 76%. (OECD, Society at a Glance, 2012). It even exceeds the average of the European Union countries, which is 67%. Borrell et al., (2001) in their study argue that deterioration of the subjective assessments of health level is a result of anxiety induced by failure to fulfill family, social and economic roles due to economic deprivation. Health self-assessment as perceived by the people themselves contributes to the effort of determining the health level of the population and is considered an important indicator of this, as it can reflect diseases which have not yet been diagnosed by medical examinations and thus constitutes a predictor of hospital and out-of-hospital health services use (McKeen, Chipperfield, & Campell, 2004)

In addition, the results of this research showed that the unemployed had more physical complaints and impaired social functioning. Similar findings are reported in the study of Hammarstrom & Janlert, (2003), which showed that the relationship between employment and unemployment affects the subjective perception of physical and mental health in a different way. Job loss increases the risk of psychiatric disorders and their physical impact, bringing forward a strong relevance between unemployment, increased depression and anxiety, and impaired social interaction. A research of Bouras and Lykouras, (2011) indicates that the negative effects of unemployment are more obvious in long-term unemployed (> 6 months) in relation to unemployment of a few months, while it has been found that the unemployed suffer from low self-esteem and strong feelings of social isolation and deprivation compared to active employees (Rantakeisu & Jonsson, 2003).

According to Kyriopoulos & Tsiantou, (2010), the looming deterioration of health indicators due to the economic crisis, with main risk factors the unemployment, the threatened work and a large decrease in income, leads to an increase in the incidence of diseases such as anxiety, depression, heart diseases and chronic diseases. The above are also confirmed by the OECD research in 2012 at global level, which showed that the economic crisis has led to an increase in poverty, unemployment and anxiety, which are associated with worse health outcomes (OECD Health at a Glance, 2012).

Smoking dependence is a major Public Health problem associated with mood disorders and established cardiovascular or pulmonary disease. (Maritz & Mutemwa, 2012; Michal et al., 2013) Smoking dependence was also associated with mood disorders in the present work. Similarly, the relation between depression and chronic diseases, physical or otherwise, was also demonstrated in the studies of Clyde, Smith, Gariépy, and Schmitz (2013) and Koulouri, Roupa, Sotiropoulou, & Skopelitou (2009). Chronic physical illnesses and comorbidity burden the individual primarily with organic and functional problems, frequent hospitalizations, continuous medical visits and examinations, and secondarily with social, psychological and occupational problems (Koulouri, Roupa, Sotiropoulou, & Skopelitou 2009). Parameters such as low income, insecurity, hopelessness, social change, social exclusion, as well as comorbidity with physical diseases affect both the emotional and mental health of individuals (Patel & Kleinman, 2003). In addition, the research of Katon, Lin, and Kroenke, (2007) showed that anxiety, depression and physical complaints are associated with the existence of chronic diseases such as diabetes, pulmonary disease, heart diseases and arthritis. Beekman et al. (2000), in their research on COPD and mood disorders, indicate that their coexistence leads to higher levels of functional limitations.

In conclusion, the present study confirms that poor socioeconomic conditions are associated with increased physical and mental morbidity and shows that in industries with increased requirements, such as shipbuilding, the deterioration of health may be greater than that of the general population, at least as reflected in health self-assessment. Indeed, the health level of an individual, as determined by self-assessment, shows strong correlation on mortality, beyond and regardless of risk factors associated with the individual’s medical history and the way of life he/she follows (Idler & Benyamini, 1997). The socio-economic context is disrupted when there is a job loss and displacement from the labor market, thus contributing to the reduction of individual expectations and the adoption of health-damaging behaviours (Toundas, 1999). Conversely, health can be improved through implementing a more equitable economic policy (De Vogli, 2014; Hanandita, & Tampubolon, 2014). In our country, the looming deterioration of health indicators due to economic crisis, with unemployment being the main risk factor, causes increased incidence of mood disorders (Kyriopoulos & Tsiantou, 2010). An epidemiological research to investigate the socioeconomic characteristics of specific employment sectors from a much wider geographical area would be useful in order to reach more general and valid conclusions.
